# Endoplasmic reticulum stress orchestrates tumor metabolism and immunity: new insights into immunometabolic therapeutics

**DOI:** 10.3389/fimmu.2025.1674163

**Published:** 2025-09-30

**Authors:** Zhang Fu, Mengyue Li, Huaixiang Zhou, Xin Zhong, Zhiqiang Zhang, Xianwen Meng, Youheng Jiang, Tao Wang, Ningning Li

**Affiliations:** ^1^ Tomas Lindahl Nobel Laureate Laboratory, The Seventh Affiliated Hospital of Sun Yat-sen University, Shenzhen, China; ^2^ Department of Geriatrics, The Seventh Affiliated Hospital of Sun Yat-sen University, Shenzhen, China; ^3^ Digestive Diseases Center, Guangdong Provincial Key Laboratory of Digestive Cancer Research, The Seventh Affiliated Hospital of Sun Yat-sen University, Shenzhen, China; ^4^ Future Medical Center, Shenzhen University of Advanced Technology, Shenzhen, China

**Keywords:** endoplasmic reticulum stress, unfolded protein response, metabolic reprogramming, tumor immune microenvironment, immunometabolism

## Abstract

Endoplasmic reticulum (ER) stress and its adaptive signaling network have emerged as central regulators of tumor progression, metabolic rewiring, and immune modulation. Within the nutrient-deprived and hypoxic tumor microenvironment, ER stress reprograms glucose, lipid, and amino acid metabolism, exerting context-dependent effects that influence both tumor cell viability and immune regulation. Concurrently, ER stress remodels the metabolic fitness and functional states of immune cells, influencing T cell exhaustion, macrophage polarization, and dendritic cell maturation. Emerging evidence indicates that tumor- and immune-cell-derived metabolites (e.g., lactate, fatty acids, and tryptophan derivatives) exert both metabolic and immunomodulatory functions, thereby shaping a dynamic “ER stress–metabolism–immunity” axis that underlies cancer heterogeneity, immune evasion, and therapeutic resistance. In this review, we synthesize recent advances delineating how canonical ER stress pathways intersect with immunometabolic reprogramming across tumor and immune compartments, and we discuss how this integrated axis reshapes the tumor immune microenvironment (TIME). Targeting this integrated axis may unveil new strategies to overcome metabolic vulnerabilities and enhance the efficacy of immunotherapy.

## 1 Introduction

The Endoplasmic reticulum (ER) is a critical organelle in eukaryotic cells that governs protein folding, lipid biosynthesis, and calcium homeostasis. Perturbations such as hypoxia, nutrient deprivation, and oxidative stress disrupt ER function and lead to the accumulation of misfolded proteins, which in turn activates the unfolded protein response (UPR) (1). This highly conserved signaling network consists of three transmembrane sensors: protein kinase RNA-like ER kinase (PERK), inositol-requiring enzyme 1 alpha (IRE1α), and activating transcription factor 6 (ATF6) (2). These sensors initiate transcriptional and translational programs that govern cell fate decisions ranging from adaptive survival to apoptosis. Persistent ER stress is a central regulator of cancer hallmarks, influencing cell proliferation, apoptosis resistance, epithelial–mesenchymal transition, and genomic stability (3). Beyond tumor-intrinsic effects, ER stress also reprograms immune cell metabolism and modulates their functional fate (4). ER stress acts as a central hub linking tumor metabolism and immune regulation via metabolite-mediated crosstalk, a key axis underlying cancer progression (5).

Metabolic reprogramming is a hallmark of cancer that enables malignant cells to survive and proliferate under hypoxic and nutrient-limited conditions. Increasing evidence identifies ER stress as a central upstream regulator of this process, integrating extrinsic stress signals with transcriptional control of metabolic networks. The PERK–ATF4 axis enhances glycolysis through GLUT1 and HK2 induction, IRE1–XBP1 promotes lipid desaturation and membrane synthesis, and ATF4 supports amino acid uptake and one-carbon metabolism (6–8). These metabolic changes support tumor cell survival by maintaining redox balance, meeting anabolic demands, and sustaining growth under stress. In parallel, metabolic by-products such as lactate, kynurenine, and fatty acids produced suppress CD8^+^ T cell cytotoxicity, promote regulatory T cell expansion, and drive M2-like macrophage polarization. In addition, depletion of key amino acids like arginine, glutamine, and serine limits T and NK cell effector function (9). ER stress–induced metabolic rewiring facilitates tumor adaptation while reshaping immune responses through the redistribution of immunomodulatory metabolites.

Within the tumor immune microenvironment (TIME), immune cells undergo extensive metabolic remodeling to sustain their activation, differentiation, and effector functions. For instance, effector T cells and natural killer (NK) cells rely on glycolysis for rapid energy supply, whereas regulatory T cells (Tregs) and alternatively activated (M2-like) macrophages preferentially utilize fatty acid oxidation and oxidative phosphorylation (10, 11). The availability of key amino acids, including arginine, glutamine, and tryptophan, plays a crucial role in shaping immune cell fate and functional polarization (12). ER stress, particularly through the PERK–ATF4 and IRE1–XBP1 axes, modulates immune cell metabolism, influencing T cell exhaustion, macrophage polarization, and dendritic cell maturation. These immunometabolic adaptations reshape immune function while concurrently driving malignant traits including epithelial–mesenchymal transition (EMT), immune evasion, stemness, and metastatic dissemination.

ER stress plays dual roles in tumor and immune cells by reprogramming metabolism and promoting their crosstalk via metabolite signaling. Key metabolites such as lactate, lipids, and amino acid derivatives drive immune evasion and tumor heterogeneity. Understanding the integrated axis of “ER stress–metabolism–immunity” provides critical insights into tumor biology and may inform strategies to overcome resistance and enhance immunotherapy efficacy.

## 2 Methods and search strategy

A systematic literature search was conducted in PubMed using keywords including “endoplasmic reticulum stress”, “UPR”, “immune microenvironment”, “metabolism”, “glucose metabolism”, “lipid metabolism”, “amino acid metabolism”, and “cancer” to retrieve English articles published from January 2009 to June 2025. The final literatures included for in-depth analysis were selected based on strict criteria: (1) high relevance to the research theme; (2) rigorous experimental design; (3) complete and reliable data. To ensure objectivity, a double-blind screening process was adopted, where two researchers independently evaluated literature quality, with discrepancies resolved through expert group discussions. In the comprehensive analysis, we paid special attention to the mutual verification between different studies, incorporating both supporting evidence and opposing viewpoints to ensure the scientificity and reliability of conclusions. Additionally, key foundational studies published before 2010 that have significant impacts on the development of this field in terms of theories or methods were also included as appropriate to provide a complete historical context.

## 3 ER stress coordinates metabolic reprogramming in cancer cells

### 3.1 ER stress coordinates glucose metabolic reprogramming in cancer cells

Metabolic reprogramming is a key adaptation that enables cancer cells to sustain proliferation and survival under stress. A hallmark of this adaptation is aerobic glycolysis, or the Warburg effect, which enables ATP generation and macromolecule synthesis even in oxygen-rich conditions (13). Emerging evidence supports a role for ER stress in this reprogramming, which can promote glycolytic enzyme expression, glucose uptake, and metabolic flexibility in defined tumor models and stress contexts (14). The following sections examine how the three canonical ER stress pathways modulate glucose metabolism in cancer.

#### 3.1.1 PERK–eIF2α–ATF4 axis promotes glycolysis and metabolic adaptation under ER stress

The PERK–eIF2α–ATF4 signaling axis is a central regulator of glycolytic reprogramming in cancer under ER stress. Upon stress sensing, PERK phosphorylates eIF2α, promoting selective translation of ATF4, which in turn activates key glycolytic enzymes such as hexokinase 2 (HK2) (15), pyruvate dehydrogenase kinase 1 (PDK1) (16), pyruvate kinase M2 (PKM2) (17), and lactate dehydrogenase A (LDHA) (18), which has been shown to increase glycolytic flux and sustain lactate production and ATP generation in specific cell systems. ATF4 further enhances glycolysis and ATP production by repressing TIGAR through CHOP-mediated transcriptional regulation (19).

Tumor-specific adaptations of this pathway are evident across cancer types. The PERK-ATF4 pathway regulates HK2 through tissue-specific mechanisms. In glioma, PERK activation promotes mitochondrial translocation of HK2 via the AKT pathway, augmenting glucose phosphorylation and preventing apoptosis by inhibiting cytochrome c release (20). In colorectal cancer, the ATF4-SLC1A5 axis promote glycolytic enhancement by driving HK2 and PKM2 expression (15). In squamous carcinoma, ATF4 overexpression rescue METTL1-dependent tumor suppression via glycolysis induction (19). Moreover, during endoplasmic reticulum stress, the PERK/eIF2α/ATF4/CHOP signaling pathway contributes to tumor progression by modulating HIF-1 expression (20). HIF-1α further enhances glycolysis by inhibiting the conversion of pyruvate to acetyl-CoA and upregulating the expression of glucose transporters and glycolytic enzymes (21–24). The PERK cofactor BZW1 further strengthens this loop by enhancing eIF2α phosphorylation and promoting IRES-dependent translation of HIF-1α and c-Myc, reinforcing the Warburg effect (25). In cancer-initiating cells (CICs), a GRP78–PERK–NRF2 axis upregulates LDHA and PDK1, shifting metabolism toward aerobic glycolysis (16).

Beyond glycolysis, the PERK–ATF4 axis supports metabolic adaptation under nutrient stress. ATF4 promotes the expression of various nutrient transporters, including SLC1A5 and GLUT5, enabling alternative substrate utilization (26). In glucose-deprived glioblastoma, ATF4 induces the expression of GLUT5 and ALDOB to support fructose metabolism, which contributes to poor prognosis in glioblastoma (26). In non-small cell lung cancer, ATF4 upregulates PCK2, allowing continued mitochondrial function and survival under glucose starvation by minimizing ROS accumulation (27).

#### 3.1.2 IRE1–XBP1 axis reinforces hypoxia-adaptive glycolytic signaling

The IRE1α–XBP1 axis promotes glycolysis in hypoxic, ER-stressed tumors. Upon activation, IRE1α catalyzes the splicing of XBP1 mRNA to generate XBP1s, a transcription factor that regulates HK2 expression (28), and cooperates with HIF-1α to induce glycolytic enzymes such as LDHA (3, 29), thereby reinforcing glycolytic flux. In addition, XBP1 upregulates PDK1, thereby blocking pyruvate entry into the TCA cycle, functionally shifting metabolism toward aerobic glycolysis in non-small cell lung cancer cells (30). In parallel, the IRE1–XBP1 axis promotes metabolic adaptation through increased glucose uptake and enhanced flexibility. XBP1s upregulates GLUT1, facilitating glucose transport into cells (3), and suppresses FOXO1, further supporting nutrient uptake and survival under metabolic stress (31). Together, these mechanisms enable cancer cells to sustain energy production and proliferation in hypoxic, nutrient-limited microenvironments. PERK-ATF4 and IRE1α-XBP1 synergistically upregulate glycolysis and glucose uptake under hypoxia/glucose deprivation, thereby maintaining energy supply and lactate output.

#### 3.1.3 ATF6: a potential modulator of glycolytic reprogramming

ATF6 has been less studied in glycolysis, but emerging evidence suggests a potential role in tumor metabolic reprogramming. In pancreatic ductal adenocarcinoma (PDAC), ATF6 activation enhances mTOR signaling (32), which promotes glucose uptake and stabilizes HIF-1α (33), thereby upregulating glycolytic enzymes such as HK2 and GLUT1 (34). In select tumor contexts (e.g., PDAC), ATF6 activation has been linked to mTOR signaling and HIF-1α stabilization and can, under sustained ER stress, indirectly augment glycolysis; this relationship is context- and duration-dependent (31, 32). The role of ATF6 in glycolysis is mostly an indirect contribution mediated through the mTOR-HIF-1α pathway.

Together, these findings establish ER stress signaling as a critical regulator of glucose metabolic reprogramming in cancer, with each UPR branch contributing uniquely to the enhancement of glycolysis and adaptation to microenvironmental stress.

### 3.2 ER stress reprograms lipid metabolism to support tumor growth

Lipid metabolic reprogramming is a hallmark of cancer, sustaining membrane biosynthesis, redox homeostasis, and oncogenic signaling. Accumulation of FFAs induces ER stress through lipid overload, oxidative stress, and proteostatic imbalance. In response, ER stress triggers metabolic adaptations that reshape lipid metabolism and link ER function to immunometabolic remodeling.

#### 3.2.1 PERK–eIF2α–ATF4 axis: coordinating lipid redistribution under metabolic stress

The PERK–eIF2α–ATF4 branch orchestrates a coordinated response to metabolic and oxidative stress by regulating fatty acid synthesis, oxidation, and storage. Through the transcriptional induction of C/EBP and SREBP1/2, this axis enhances *de novo* lipogenesis and cholesterol synthesis to support ER membrane biogenesis and lipid turnover (35). Under glutamine deprivation, ATF4 upregulates the E3 ubiquitin ligase TRIM2, which stabilizes and activates CPT1A—the rate-limiting enzyme of mitochondrial fatty acid β-oxidation—thereby facilitating lipid catabolism and preventing apoptosis (36). Simultaneously, ATF4 promotes lipid droplet formation, sequestering free fatty acids to mitigate lipotoxicity and preserve ER integrity (37). Together, these mechanisms confer metabolic resilience by sustaining lipid homeostasis and adaptability under nutrient-limited conditions. IRE1α-XBP1 potently drives lipogenesis and ER membrane expansion, primarily through the SCD1/DGAT/LPCAT/sterol synthesis axis.

#### 3.2.2 IRE1–XBP1 axis: promoting lipid synthesis and ER membrane expansion

The IRE1–XBP1 pathway is a key driver of lipid metabolic reprogramming, coupling lipogenesis with ER membrane expansion and cellular adaptation. Upon ER stress, spliced XBP1 (XBP1s) transcriptionally activates a panel of lipid-metabolic enzymes—including SCD1, DGAT2, LPCAT3, FASN, ACLY, ACC1, HMGCR, and HMGCS1—which is consistent with facilitating the synthesis of unsaturated fatty acids, triacylglycerols, phospholipids, and sterols (37). These lipid species support ER membrane remodeling and maintain secretory function under stress.

In cancer, this axis plays a pivotal role in metabolic adaptation. In MYC-driven tumors, XBP1-driven upregulation of SCD1 enhances membrane fluidity and unsaturated lipid content, preserving ER homeostasis and promoting proliferation (38). In hepatocellular carcinoma, the IRE1–SEC63–ACLY signaling cascade links ER stress to lipid rewiring and metastatic progression by increasing acetyl-CoA production and histone acetylation, thereby integrating lipid metabolism with transcriptional regulation of UPR targets and metastasis-related genes (39). PERK-ATF4 supports fatty acid oxidation (FAO) and lipid droplet buffering to maintain lipid homeostasis under nutrient deprivation.

#### 3.2.3 ATF6 axis: preserving membrane lipid composition and ER structure

Although less well-characterized in oncogenesis, the ATF6 branch contributes significantly to ER membrane maintenance during stress. ATF6 activation induces transcription of genes involved in phospholipid biosynthesis, including CHKA, PCYT1A and LPIN1, as well as genes regulating lipid droplet formation such as PLIN2, DGAT1, DGAT2, FITM1 and FITM2. This coordinated gene regulation ensures optimal phospholipid composition and facilitates safe lipid storage under endoplasmic reticulum stress conditions (37). These actions buffer changes in membrane tension and facilitate ER membrane expansion, preserving protein folding capacity under stress. Despite its essential role in ER lipid homeostasis, the involvement of ATF6 in tumor-associated lipid remodeling remains largely unexplored. ATF6 mainly preserves the phospholipid composition and structural integrity of the ER membrane, though evidence for its direct role in tumor lipid remodeling remains limited.

#### 3.2.4 Other UPR regulators: linking lipid remodeling to immune evasion

Beyond the canonical UPR branches, emerging regulators such as GRP78 and P4HB modulate lipid metabolism with implications for tumor immune evasion. GRP78 inhibition disrupts lipid homeostasis, leading to the accumulation of free fatty acids and downregulation of SREBP1 and CPT1A, collectively impairing lipogenesis and mitochondrial fatty acid oxidation (38). Integrative multi-omics analyses reveal that ER stress markers, particularly P4HB, co-express with key lipid metabolic genes such as APOE and NPC1L1, showing significant correlation with diminished CD8^+^ T-cell infiltration and inferior immunotherapy outcomes (39). These findings underscore a critical intersection between ER stress–induced lipid rewiring and tumor immunometabolism.

Together, these findings establish ER stress signaling as a central regulator of lipid metabolic reprogramming in cancer, with each UPR branch uniquely orchestrating fatty acid synthesis, oxidation, and storage to sustain membrane integrity, redox balance, and survival under metabolic stress.

### 3.3 ER stress reprograms amino acid metabolism in cancer cells

In nutrient-deprived tumor microenvironments, adaptive remodeling of amino acid metabolism is essential for cancer cell survival. ER stress activates the UPR to regulate amino acid uptake, biosynthesis, and processing of amino acid–derived metabolites, thereby rewiring metabolic networks to sustain growth and promote cellular adaptation under stress conditions.

#### 3.3.1 PERK–eIF2α–ATF4 axis: coordinating amino acid uptake, biosynthesis, and redox balance

The PERK–eIF2α–ATF4 arm of the UPR serves as a central regulator of amino acid metabolism in tumor cells under stress (14). ATF4 transcriptionally activates a suite of genes involved in amino acid transport (e.g., SLC1A5, SLC7A5) (40, 41), biosynthesis (ASNS) (42), and sulfur metabolism (CTH, GCL) (43), which is consistent with increasing amino acid uptake and biosynthetic capacity.

Under glucose deprivation, ATF4 promotes glutamine flux into the hexosamine biosynthetic pathway via GFAT1, maintaining cysteine levels and facilitating glutathione (GSH) synthesis to buffer oxidative stress and enhance radio resistance (43). In response to glutamine or methionine scarcity, ATF4 integrates signals from EIF2AK4 (GCN2) to activate the AKT–mTORC2 axis, preserving metabolic homeostasis (44). In KRAS-mutant non-small-cell lung cancer (NSCLC), ATF4-driven ASNS expression is essential for survival under nutrient restriction (42). Simultaneously, ATF4 upregulates DDIT4 (REDD1), which inhibits mTORC1 activity and induces cytoprotective autophagy (45).

ATF4 also cooperates with CHOP to regulate genes involved in amino acid biosynthesis and autophagic recycling, further promoting survival under nutrient-deprived conditions (45). Additionally, ATF4 upregulates SLC7A5 to enhance leucine uptake and sustain mTOR signaling (41). In melanoma, LDHA inhibition activates the EIF2AK4–ATF4 cascade, driving serine and aspartate biosynthesis and SLC1A5 expression, thereby augmenting glutamine and essential amino acid uptake to support mTOR-mediated proliferation (46). Moreover, IDO/TDO-mediated tryptophan catabolism activates ATF4-dependent upregulation of SLC1A5 and its splice variants, facilitating compensatory uptake of glutamine and tryptophan to maintain cellular fitness (41). PERK-ATF4 coordinates amino acid uptake, biosynthesis, and antioxidant defense by regulating the SLC1A5/SLC7A5 transporters, ASNS synthetase, and GSH antioxidant system.

#### 3.3.2 IRE1–XBP1 axis: modulating amino acid metabolism and antigen presentation

Although less studied than PERK, the IRE1–XBP1 branch contributes to amino acid–related metabolic remodeling, particularly under hypoxic or lipotoxic conditions. In B16 melanoma cells, IRE1 activation and XBP1 expression promote tyrosinase induction and melanin production during α-MSH–stimulated melanogenesis, suggesting a role in amino acid–derived pigment biosynthesis (47). In parallel, XBP1 overexpression impairs MHC-I antigen presentation by disrupting peptide loading, facilitating tumor immune evasion (48). These findings underscore the involvement of the IRE1–XBP1 axis in both metabolic adaptation and immunomodulation during ER stress.

#### 3.3.3 ATF6 axis: facilitating amino acid–dependent protein folding and melanogenesis

ATF6, though less extensively characterized in amino acid metabolism, is essential for ER proteostasis and amino acid–dependent biosynthetic processes. In B16 melanoma cells, ATF6 inhibition suppresses tyrosinase expression and melanin synthesis in response to α-MSH, whereas chemical chaperones rescue both phenotypes—indicating a role in pigment production and ER folding capacity (47).

ATF6 also cooperates with XBP1 to impair MHC-I surface expression under ER stress, compromising antigen presentation (49). As a transcriptional activator of ER chaperones and ER-associated degradation (ERAD) machinery, ATF6 likely contributes indirectly to amino acid metabolic adaptation by maintaining ER folding homeostasis during nutrient stress (50).

**Figure 1:**
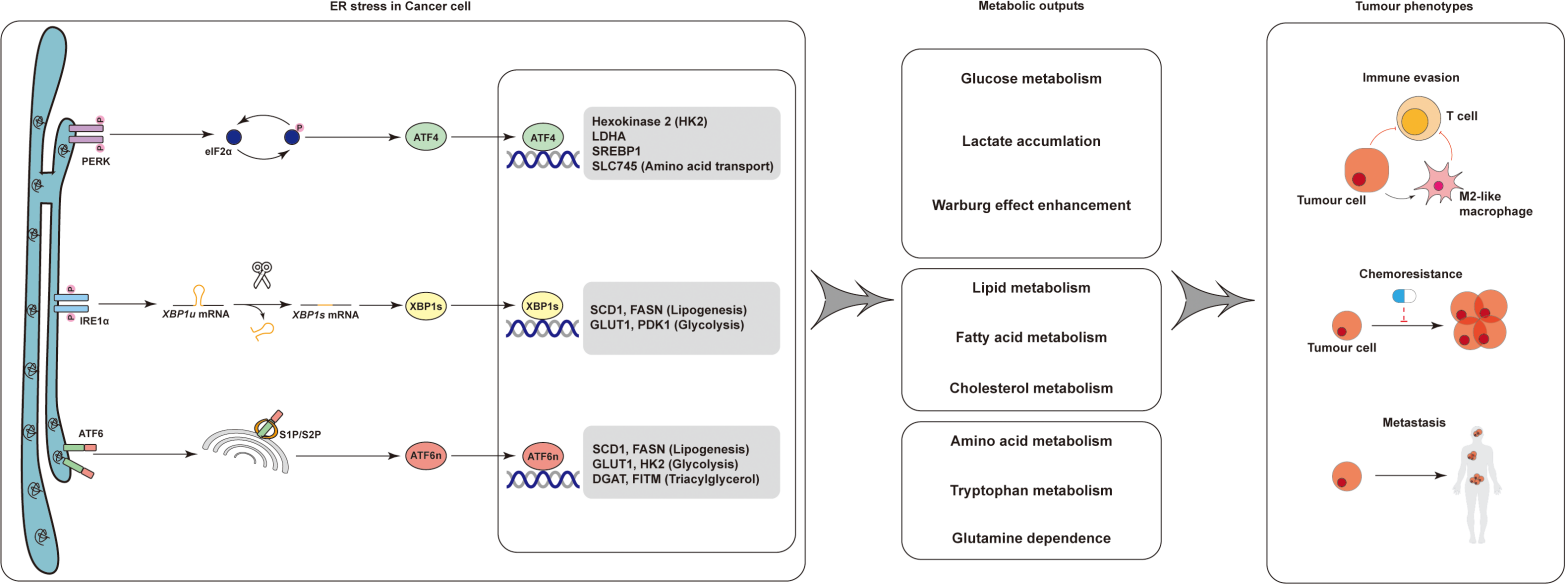
Schematic of ER stress-mediated regulation of tumor metabolism and phenotypes. ER stress in cancer cells activates three canonical unfolded protein response (UPR) branches: PERK–eIF2α–ATF4, IRE1α–XBP1s, and ATF6. These signaling axes orchestrate transcriptional programs that reshape glucose, lipid, and amino acid metabolism. The resulting metabolic reprogramming—characterized by enhanced aerobic glycolysis (Warburg effect) and aberrant lipid/amino acid utilization—reinforces tumor hallmarks such as immune evasion, chemoresistance, and metastasis. These insights highlight ER stress as a central hub for targeting tumor immunometabolism and therapeutic resistance.

Together, these branches of the unfolded protein response reprogram amino acid metabolism to support tumor growth, redox balance, and immune evasion under endoplasmic reticulum stress (**Figure 1**).

## 4 Metabolites shape immune cell function in the tumor microenvironment

### 4.1 Glycolytic metabolites shape immune cell function in the tumor microenvironment

Within the nutrient-deprived TIME, glycolysis emerges as a central regulator of immune cell fate and function. M1-like macrophages preferentially engage glycolysis, with intermediates such as succinate reinforcing classical polarization. In contrast, M2-like macrophages rely on oxidative phosphorylation (OXPHOS) to support anti-inflammatory programs (51, 52). Myeloid-derived suppressor cells (MDSCs) shift toward glycolysis under hypoxia, leading to lactate accumulation and ROS reduction, which enhance their immunosuppressive capacity (53). Tregs exploit lactate and fatty acids to sustain suppressive functions through mitochondrial metabolism (54).

#### 4.1.1 Glycolytic metabolites potentiate antitumor immunity

Glycolytic by-products exert immunomodulatory effects in a context- and cell type-dependent manner. In CD8^+^ T cells, tumor-derived lactate inhibits pyruvate carboxylase and activates pyruvate dehydrogenase, leading to decreased succinate secretion and attenuation of SUCNR1-dependent cytotoxic signaling (55). In neuroblastoma, glycolysis-associated lipids—such as lactosylceramide and ganglioside-GD3—drive the upregulation of PD-1 and CD52 on CD8^+^ T cells, fostering an exhausted phenotype (56). In triple-negative breast cancer, aberrant d-xylose metabolism impairs immunoproteasome function via dihydrodiol dehydrogenase (DHDH), compromising antigen presentation, whereas d-xylose supplementation restores CD8^+^ T cell effector function and enhances immunotherapy responsiveness (57). Similarly, in glioma, tumor-derived d-2-hydroxyglutarate (d-2HG) is taken up by CD8^+^ T cells, suppressing cytotoxicity and impairing interferon-γ (IFN-γ) signaling (58). In pancreatic ductal adenocarcinoma (PDAC), tumor and NK cells compete for vitamin B6, ultimately dampening NK cell–mediated cytotoxicity (59). In lymphoma and melanoma, elevated pyruvate levels enhance IL-12–responsive CD8+ T cell programs and augment antitumor potential (60). A high-glucose environment activates the mTOR–FOXM1 pathway, increasing transcription of the costimulatory molecule CD27 in tumor-infiltrating CD8^+^ T cells, thereby unleashing their cytotoxic potential (61). Succinate, a glycolytic intermediate, reinforces classical macrophage polarization by promoting glycolytic flux and inhibiting the TCA cycle (62). Metabolites such as lactate and d-2HG generally inhibit the metabolic activity and effector functions of CD8^+^T cells; however, in a few specific contexts, exogenous or low-dose lactate can enhance the adaptability of CD8^+^T cells. The net effect depends on the source, dose of the metabolites, and the type of recipient cells.

#### 4.1.2 Glycolysis-driven immunosuppression in the tumor immune microenvironment

Beyond T cells, glycolytic programming plays a pivotal role in shaping the functional polarization of innate immune populations. M1-like macrophages preferentially engage glycolysis to support pro-inflammatory activity and NADH production, whereas M2-like macrophages rely on oxidative metabolism via the tricarboxylic acid (TCA) cycle (63). Myeloid-derived suppressor cells (MDSCs), under hypoxia and nutrient restriction, undergo a glycolytic shift that reduces intracellular reactive oxygen species (ROS), enhances survival, and augments their immunosuppressive phenotype (64). This metabolic adaptation enables MDSCs to generate immunosuppressive lactate and deplete local glucose, thereby constraining CD4^+^ T cell metabolism and effector function (65). In cervical cancer, the NAT10–ac4C–FOXP1 axis enhances glycolysis in tumor cells and promotes Treg-mediated immunosuppression through lactate accumulation (66).

#### 4.1.3 Tumor-derived glycolysis metabolites trigger ER stress–dependent immune regulation

ER stress contributes to this immunometabolic reprogramming. The PERK–eIF2α–ATF4 and IRE1α–XBP1 arms of the UPR regulate glycolytic gene expression and promote export of glucose-derived metabolites, shaping the immunosuppressive metabolite pool within the TIME. This ER–glycolysis axis creates a feedforward loop linking tumor stress signaling, metabolite secretion, and immune dysfunction.

### 4.2 Lipid-derived metabolites regulate immune cell function in the tumor microenvironment

Reprogrammed lipid metabolism within the TIME exerts profound immunomodulatory effects, not only supporting tumor cell adaptation but also actively suppressing anti-tumor immunity. Lipid species—including fatty acids, bile acids, and cholesterol—can influence immune cell fate, polarization, and effector function through metabolic, transcriptional, and epigenetic mechanisms.

#### 4.2.1 Fatty acid–mediated immune reprogramming

Elevated levels of free fatty acids (FFAs) in the TIME drive fatty acid oxidation (FAO) in NK cells, resulting in the upregulation of TGF-β1 and NKG2D ligands and consequent suppression of cytotoxic activity (67). In the lung metastatic niche, lipids secreted by stromal cells are directly taken up by NK cells, impairing their effector function and promoting metastatic colonization. Similarly, in adipocyte-rich environments such as breast cancer, lipid vesicles released from adipocytes serve a dual role—delivering fatty acids to tissue-resident macrophages and inducing bone marrow–derived progenitors to differentiate into immunosuppressive macrophage-like cells.

Short-chain fatty acids (SCFAs) exert context-dependent effects on immune responses. Under glucose-deprived or stress conditions, microbiota-derived acetate enhances histone acetylation in tumor-infiltrating CD8^+^ T cells via an ACSS2-dependent pathway, thereby strengthening anti-tumor activity. Intriguingly, blockade of acetate uptake by tumor cells increases acetate bioavailability for immune cells, where it is oxidized via ACSS1 to support mitochondrial respiration and T cell effector function (68, 69). Butyrate can augment CAR-T activity by engaging mTOR to increase cytokines and by HDAC inhibition to upregulate antigen-processing/presentation genes (70);in colorectal cancer, butyrate and propionate induce DNA-damage–associated antigenic remodeling, consistent with enhanced CD8^+^ responses (71).

Accumulation of unsaturated fatty acids (UFAs) in FABP5^+^ lipid-laden macrophages impair T cell–mediated immunity in hepatocellular carcinoma (HCC). Mechanistically, UFAs activate the PPARγ signaling axis, upregulating multiple immunosuppressive ligands and facilitating T cell dysfunction, highlighting a pivotal role for tumor-associated macrophages (TAMs) lipid metabolism in immune evasion (72).

#### 4.2.2 Cholesterol and bile acids mediators in immune suppression

Cholesterol depletion in the TIME represents an emerging immune checkpoint. Tumor cells and TAMs compete for cholesterol uptake, depriving CD8^+^ T cells of a critical membrane constituent and accelerating their functional exhaustion. In glioblastoma, TAMs engulf cholesterol-rich debris and adopt a lipid-laden macrophage phenotype, subsequently transferring cholesterol to tumor cells, which fuels their high metabolic demand (73). Consistent with this notion, we previously reported that IDH-mutant glioblastoma cells suppress the M1 polarization of tumor-associated microglia through the release of cholesterol (74). In colorectal cancer, tumor cells upregulate the transcription factor USF1, which activates SREBF2 and promotes the release of desmosterol, a cholesterol intermediate, into the TIME. Desmosterol uptake by CD8^+^ T cells impair the mevalonate pathway and KRAS activity, contributing to immune evasion (75).

In HCC, bile acid metabolism also contributes to immune suppression. Loss of AKR1D1 enhances the microbial conversion of primary bile acids into iso-LCA, a secondary bile acid that accumulates in hepatic and intestinal compartments and potently inhibits NK cell cytotoxicity (76). Moreover, bile acids signal through the nuclear receptor FXR, broadly expressed in monocytes and macrophages, to drive M2-like macrophage polarization and reinforce immunosuppressive TIME conditions (77).

#### 4.2.3 Tumor-derived lipids trigger ER stress–dependent immunosuppressive reprogramming in TAMs

Persistent ER stress in tumor cells promotes the synthesis and export of lipid metabolites, leading to progressive lipid accumulation within the TIME. This lipid-rich milieu, together with unresolved UPR signaling and lipid peroxidation stress, drives TAMs toward an M2-like immunosuppressive phenotype. Tumor-derived glucosylceramides remodel the lipid composition and saturation of the ER membrane in TAMs, triggering a non-canonical ER stress response characterized by IRE1-mediated XBP1 splicing and STAT3 phosphorylation. This lipid-induced UPR enhances TAMs pro-tumorigenic activity and survival, which can be reversed by genetic ablation of XBP1 or LPCAT3-mediated phospholipid remodeling. In parallel, glucosylceramides engage the pattern recognition receptor Mincle (macrophage-inducible C-type lectin), cooperatively activating IRE1α–XBP1s and IRE1α–STAT3 pathways to drive ER lipid remodeling and the transcription of immunosuppressive gene programs (78). Pharmacological or genetic blockade of lipid efflux or UPR signaling attenuates TAM-mediated immunosuppression and delays tumor progression, underscoring the pivotal role of the ER stress–lipid axis in shaping the immunosuppressive TIME. Cholesterol and bile acids suppress NK/T cells, while SCFAs (butyrate) show context-dependent immunostimulation. Lipid-laden TAMs emerge as metabolic gatekeepers in the TIME.

### 4.3 Amino acid metabolites orchestrate immune cell fate and anti-tumor response

#### 4.3.1 Nutrient competition and metabolic byproducts influence immunosuppression

To sustain their rapid proliferation, tumor cells aggressively scavenge amino acids such as tryptophan, arginine, lysine, aspartate, glutamine, methionine, and serine from the TIME, creating a nutrient-deprived milieu. This depletion compromises the survival, differentiation, and effector function of immune cells, thereby impairing anti-tumor immunity (79, 80).

Tryptophan metabolism represents a critical immunoregulatory node. It is primarily catabolized through the kynurenine pathway, in which indoleamine 2,3-dioxygenase (IDO1, IDO2) and tryptophan 2,3-dioxygenase (TDO) serve as rate-limiting enzymes (81). In glioblastoma, high expression of IDO and TDO correlates with poor prognosis (82, 83). The KP metabolite kynurenine (Kyn), an endogenous ligand of the aryl hydrocarbon receptor (AHR), suppresses T-cell activity and promotes regulatory T-cell (Treg) differentiation. In HER2-positive breast cancer, cancer-associated fibroblasts (CAFs) from trastuzumab-resistant patients show elevated IDO2 and TDO2, reinforcing a pro-tumor phenotype (84). Similarly, tumor-associated non-myelinating Schwann cells in pancreatic cancer exhibit high TDO2 expression, driving Kyn accumulation (85). Kyn–AHR signaling also exerts potent immunosuppressive effects in macrophages by inducing CD39 and IL-10 expression while dampening costimulatory signals and antigen presentation (86–88).

Arginine metabolism exerts dual roles in the TIME. While T cells require arginine to maintain proliferation and effector function, TAMs exploit arginine to fuel polyamine synthesis and reinforce their tumor-promoting polarization (89). In breast cancer, tumor-derived arginine drives TAMs secretion of TGF-β1, PD-L1, and IL-10, thereby accelerating disease progression (12). In hepatocellular carcinoma (HCC), lysine depletion reduces STAT3 signaling in T cells, impairing their proliferative and cytotoxic capacity and promoting immune evasion (90). Additionally, N-acetyl-aspartate in the TIME suppresses NK and CD8^+^ T-cell (91). In colorectal cancer, glutamine metabolism is reprogrammed to support tumor growth (92). CAFs overexpress glutamine synthetase (GS) to supply tumor cells and suppress apoptosis (93–95). Pharmacological inhibition of glutaminase (GLS) enhances immunoproteasome activity and MHC-I–dependent antigen presentation, boosting anti-tumor immunity (96). Serine metabolism provides one-carbon units for nucleotide synthesis, supporting both tumor and T cell proliferation. In macrophages, serine modulates IL-1β production via GSH- or SAM-dependent mechanisms, contributing to the inflammatory milieu (97). In HCC, tumor-derived 4-acetylaminobutyric acid (4-Ac-GABA), a metabolite of GABA produced by ACAT1 recruited by phosphomevalonate kinase, acts on CD8^+^ T cells through GABAA receptors, suppressing AKT signaling and limiting activation, infiltration, and cytotoxic function (98). Meanwhile, tumor-derived ammonia accumulates in the TIME and impairs NK cell cytotoxicity.

#### 4.3.2 Tumor-derived amino acid derivatives trigger ER stress–dependent immune regulation

**Figure 2:**
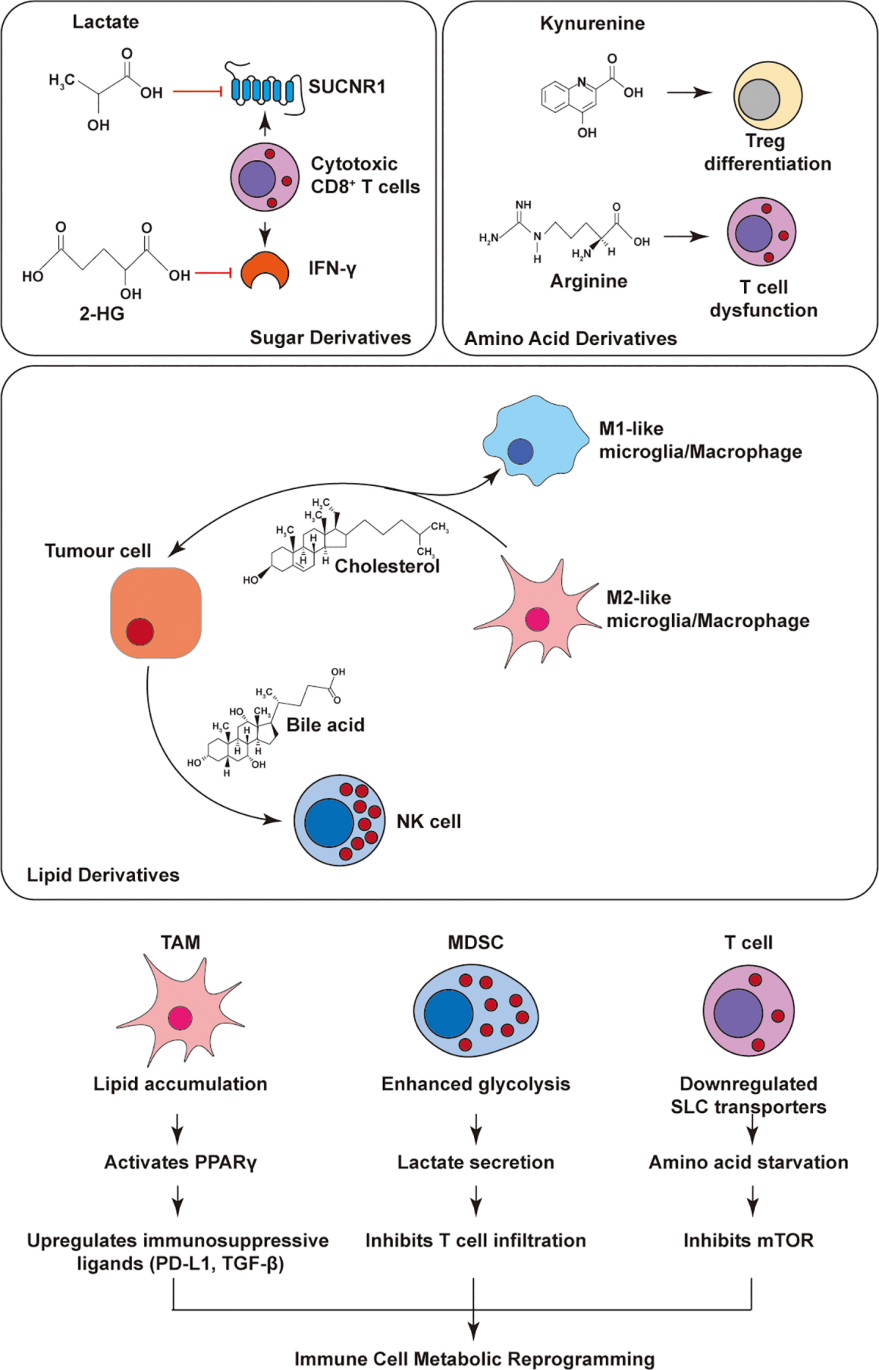
Tumor-immune metabolic crosstalk in the tumor microenvironment. Tumor-derived metabolites shape immune responses through distinct metabolic pathways. Lactate and 2-hydroxyglutarate impair CD8^+^ T cell function. Kynurenine promotes Treg differentiation, while arginine depletion suppresses T cells. Lipid metabolites such as cholesterol and bile acids modulate macrophage polarization and inhibit NK cells. Meanwhile, TAMs, MDSCs, and T cells undergo metabolic reprogramming (e.g., lipid accumulation, glycolysis, PPARγ activation), collectively contributing to immune suppression. This highlights key targets for metabolic intervention in tumor immunity. Kynurenine and arginine depletion dominate immunosuppression, with IDO/TDO inhibition restoring T cell function.

The UPR, particularly the PERK–eIF2α–ATF4 branch, is a central node linking amino acid metabolism and immune adaptation. ATF4 transcriptionally upregulates genes encoding amino acid transporters (e.g., SLC1A5, SLC7A5) (40, 41), biosynthetic enzymes (e.g., ASNS, CTH) (43), and antioxidant pathways to maintain redox balance and nutrient availability. In T cells, extracellular amino acid deprivation induces ATF4 activation, triggering a metabolic shift that enhances glycolysis, glutaminolysis, and oxidative phosphorylation to support survival under nutrient stress (99). Together, these findings highlight the ER stress–amino acid axis as a critical safeguard mechanism preserving immune competence within a hostile TIME (**Figure 2**).

## 5 ER stress regulates immune cell metabolism

Sustained ER stress reprograms immune cell metabolism to modulate activation, differentiation and exhaustion within the tumor microenvironment. This regulation is particularly important in metabolically active populations such as T cells, dendritic cells and macrophages, highlighting ER stress as a key determinant of immune cell fate and antitumor immunity.

### 5.1 ER stress shapes T cell metabolic programming

T cells rely on flexible metabolic rewiring, coordinated in part by ER stress and its adaptive UPR response, to sustain their function in hostile environments such as the tumor microenvironment. PERK–eIF2α–ATF4 signaling promotes metabolic adaptation and mitochondrial homeostasis, supporting T cell activity. In contrast, chronic IRE1–XBP1 activation impairs glucose and glutamine metabolism, driving T cell exhaustion. UPR components like GRP78 and Gp96 also regulate calcium signaling and glycolysis, linking stress sensing to immune function.

#### 5.1.1 PERK–eIF2α–ATF4 axis: promoting metabolic adaptation and antitumor functions

T cell activation demands a rapid metabolic shift, transitioning from fatty acid oxidation and oxidative phosphorylation to a glycolysis-dominant state. This metabolic reprogramming triggers ER stress due to elevated protein synthesis, thereby activating the PERK–eIF2α–ATF4 arm of the UPR. PERK-mediated phosphorylation of eIF2α temporarily suppresses protein translation while inducing ATF4, which upregulates key glycolytic genes such as GLUT1 and HK2, enhancing glucose uptake and effector function (6, 100). In melanoma models, carbon monoxide (CO) stimulation activates PERK, inducing protective autophagy and mitophagy, facilitating the clearance of damaged mitochondria and restoring metabolic homeostasis (101). Furthermore, GRP78 and ATF4 induction under ER stress promotes metabolic programs required for T cell activation (100).

#### 5.1.2 IRE1–XBP1 axis: impairing T cell metabolism and antitumor immunity

Chronic activation of the IRE1–XBP1 pathway contributes to CD8^+^ T cell exhaustion in the TIME. Chronic activation of the IRE1α–XBP1 pathway is linked to CD8^+^ T-cell exhaustion. XBP1 suppresses GLUT1 and downregulates the glutamine transporter SLC38A2, thereby restricting glucose and glutamine uptake required for mitochondrial metabolism and leading to reduced IFN-γ production and respiratory capacity (14, 102, 103). The biochemical mechanism of lactate-mediated cytotoxic-signaling suppression is not repeated here and is cross-referenced to Section 3.1 (102, 103). Beyond metabolic suppression, XBP1 also modulates Th17 polarization, amino acid metabolism (e.g., tryptophan and glutamine), and calcium signaling (103). For example, Gp96 deficiency in CD4^+^ T cells impairs calcium mobilization and glycolytic flux (100), while XBP1 splicing induced by hypoxia or glucose deprivation promotes Th17 differentiation—a process reversible by TUDCA, which attenuates disease progression in EAE models (104, 105). Acute TCR stimulation triggers PERK-ATF4 signaling to support glycolytic activation and mitochondrial quality control, whereas chronic IRE1α-XBP1 activation drives GLUT1/SLC382 downregulation, correlating with impaired IFN-γ production and restricted respiratory capacity. This dichotomy mirrors the differential metabolic adaptations to acute versus chronic ER stress under TIME conditions.

#### 5.1.3 Other UPR regulation of T cell metabolism

In addition to classical PERK and IRE1 arms, UPR regulates T cell metabolism via non-canonical mechanisms. Following TCR engagement, the ER-resident chaperone and calcium buffer Gp96 is upregulated, facilitating cytosolic calcium signaling critical for T cell activation. Gp96 deletion in CD4^+^ T cells disrupts calcium flux, leading to impaired glycolysis and early clonal expansion (100). Early T-cell activation can involve PERK–eIF2α–ATF4–associated increases in glucose uptake and mitochondrial homeostasis, whereas prolonged intratumoral stress engages IRE1α–XBP1 with reduced glucose/glutamine uptake, IFN-γ, and respiration; this pattern is consistent with Chen & Cubillos-Ruiz 2020 and supported by primary data (8, 14, 100–103).

### 5.2 ER stress shapes TAMs and MDSC cell metabolic programming

TAMs and myeloid-derived suppressor cells (MDSCs) are metabolically adaptable immune cells that support tumor immune evasion and reprogramming. TAMs shift between glycolysis-driven M1-like states and OXPHOS/FAO-dependent M2-like states, while MDSCs deploy distinct metabolic pathways to suppress T cell function. Recent studies highlight that ER stress and the UPR reprogram the metabolism of both cell types, reinforcing their immunosuppressive phenotypes and shaping the tumor microenvironment.

#### 5.2.1 PERK–eIF2α–ATF4 axis enhances M2 polarization via metabolic reprogramming

The PERK–eIF2α–ATF4 signaling pathway plays a central role in promoting immunosuppressive M2 polarization through metabolic rewiring. In response to IL-4 stimulation, PERK activation upregulates ATF4, which transcriptionally induces phosphoserine aminotransferase 1 (PSAT1), enhancing serine biosynthesis and mitochondrial α-ketoglutarate production (106). This, in turn, fuels JMJD3-dependent epigenetic remodeling to stabilize the M2 phenotype. In glioblastoma (GBM), PERK-ATF4 enhances TAM-mediated immunosuppression via GLUT1 upregulation (107). PERK also upregulates arginase 1 (ARG1), depleting extracellular arginine and thereby impairing T cell proliferation and CD3ζ expression. Genetic or pharmacological inhibition of PERK in TAMs reverses this metabolic program, restores M1 identity, and enhances antitumor immunity (108).

In addition to TAMs, PERK–ATF4 signaling also regulates myeloid-derived suppressor cells (MDSCs). Tumor-derived factors can induce GLUT1 expression in monocyte-derived MDSCs (MDMs) through PERK–ATF4 activation. This facilitates histone lactylation and reinforces immunosuppressive function. PERK deletion in MDMs disrupts lactylation, promotes T cell accumulation in the tumor, delays tumor growth, and synergizes with immune checkpoint therapy to suppress GBM progression (8).

#### 5.2.2 IRE1–XBP1 axis suppresses M1 functions and promotes immunosuppression

The IRE1–XBP1 arm of the UPR contributes to the maintenance of M2 macrophage function by favoring mitochondrial metabolism and reactive oxygen species (ROS) generation. Inhibition of this pathway reduces FAO and OXPHOS activity while enhancing glycolysis, resulting in M2-to-M1 repolarization and increased secretion of proinflammatory cytokines such as TNF-α and IL-12 (109). In GBM, XBP1 also cooperates with hypoxia-inducible factor 1α (HIF-1α) to stabilize M2-like myeloid cells and suppress antitumor responses (15).

In addition to its role in TAMs, the IRE1–XBP1 pathway also critically regulates myeloid-derived suppressor cells (MDSCs), a heterogeneous population of pathologically activated neutrophils and monocytes with potent immunosuppressive activity. MDSCs are closely associated with poor prognosis in cancer patients and are a central component of the immunosuppressive TIME (110). In lymphoblastic leukemia and triple-negative breast cancer, activation of IRE1α-XBP1 significantly increases MDSC infiltration via enhanced secretion of immunoglobulin M and tumor-derived cytokines, implicating UPR signaling in protein and lipid regulation that shapes MDSC function (111). Under hypoxic conditions, XBP1 further facilitates cholesterol biosynthesis in tumor cells by directly binding to the promoters of HMGCR and HMGCS1, leading to increased cholesterol secretion. This secreted cholesterol activates MDSCs by inducing STAT3 phosphorylation and ROS accumulation, thereby reinforcing the immunosuppressive microenvironment and supporting tumor immune evasion (7). The PERK-ATF4 axis stabilizes M2 macrophage polarization through serine/α-KG-driven metabolic reprogramming and lactate-mediated histone lactylation, while IRE1α-XBP1 sustains an immunosuppressive myeloid program by maintaining FAO/OXPHOS dependency and sterol-STAT3 activation. Notably, pharmacological inhibition of IRE1α-XBP1 promotes M2-to-M1 repolarization and synergizes with anti-PD-1 therapy. Under sustained tumor stress, UPR signaling couples to lipid/oxidative metabolism to favor OXPHOS/FAO-leaning immunoregulatory TAMs states; IRE1–XBP1 inhibition promotes M2→M1 repolarization and synergizes with anti–PD-1, with PERK–ATF4 control of serine/glucose/arginine metabolism further supporting context dependence (106–109).

### 5.3 ER stress shapes DC cell metabolic programming

Dendritic cells (DCs) are pivotal antigen-presenting cells that initiate antitumor T cell responses. However, within the TIME, persistent metabolic and proteostatic stress impairs their immunostimulatory capacity. In an orthotopic metastatic ovarian cancer model, lipid peroxidation products (e.g., 4-hydroxy-2-nonenal) form adducts with GRP78/BiP and ERdj3 in tumor-infiltrating DCs, consistent with ER stress and IRE1α-driven XBP1 splicing; this supports an XBP1s-dependent lipogenic program with cytoplasmic lipid-droplet accumulation and impaired cross-presentation, while DC-specific XBP1 deletion reduces lipid loading, restores cross-priming, and improves antitumor immunity (4). Together, these data support viewing the IRE1α/XBP1s axis as a metabolism-linked checkpoint in DCs, whereby lipid peroxidation–induced XBP1s and lipogenic remodeling contribute to immune evasion in the tumor microenvironment.

Further insights highlight the contribution of UPR to DC dysfunction. The ER-resident chaperone BAT3, involved in co-translational protein quality control, is selectively downregulated in tumor-derived DCs. BAT3 deficiency enhances IRE1α-driven transcriptional programs, reconfigures cellular metabolism, and promotes immunosuppressive glucocorticoid synthesis, ultimately dampening effective T cell activation (112).

**Figure 3:**
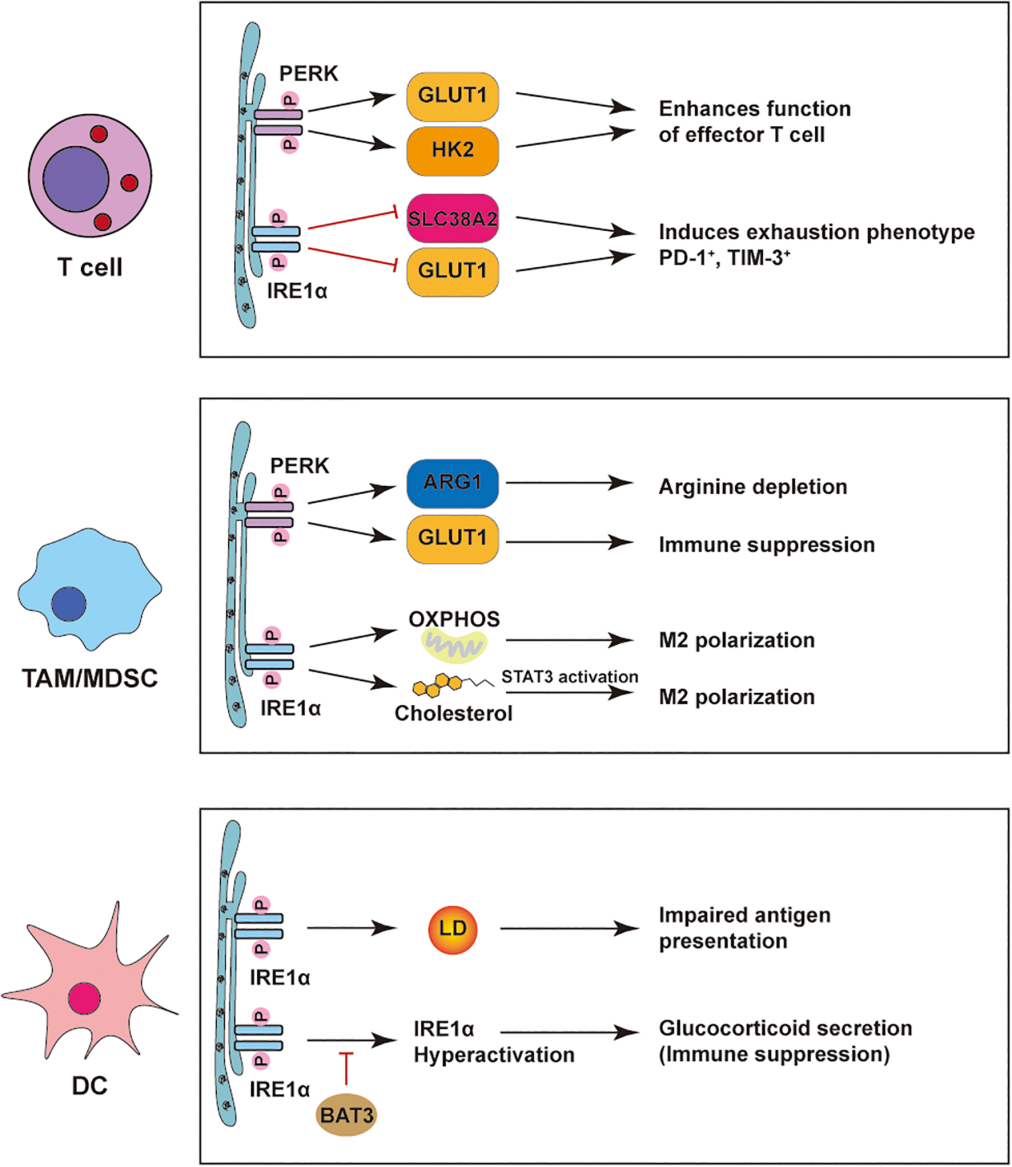
ER stress–mediated regulation of immune cells in the tumor microenvironment. PERK and IRE1α pathways reshape immune cell functions in tumors. In T cells, PERK boosts effector function via GLUT1/HK2, while IRE1α drives exhaustion through SLC38A2/GLUT1. In TAMs/MDSCs, PERK induces ARG1/GLUT1 to suppress immunity, and IRE1α promotes M2 polarization via OXPHOS, cholesterol, and STAT3. In DCs, PERK-induced lipid droplets impair antigen presentation; IRE1α hyperactivation triggers glucocorticoid-mediated suppression, relieved by BAT3.

Together, these findings position the IRE1α–XBP1s axis as a central metabolic checkpoint in DCs, whose dysregulation contributes to immune evasion. Targeting ER stress pathways in DCs may offer a promising strategy to restore antitumor immunity in metabolically hostile tumor niches. Lipid peroxidation-driven IRE1α-XBP1s activation induces lipogenic reprogramming and lipid droplet accumulation in DC cell, impairing their cross-presentation capacity. Primary data demonstrate that DC-specific Xbp1deletion reverses this metabolic blockade and restores antigen priming functionality. This pathway constitutes a critical metabolic checkpoint in DC cell, with its immunosuppressive effects modulated by both the source of lipid peroxidation products and the duration of ER stress exposure. In dendritic cells, lipid peroxidation→IRE1α–XBP1s drives lipogenic remodeling and lipid-droplet accumulation that impairs cross-presentation, as shown in Cubillos-Ruiz 2015 and aligned with Chen & Cubillos-Ruiz 2020 (4, 14) (**Figure 3**; **Table 1**).

**Table 1:** UPR arms in immune cells: context-dependent effects on antitumor immunity.

Module	Cell/context (standardized)	Key metabolic node/metabolite	Immune outcome	Ref. no.
PERK–eIF2α–ATF4	T cells (early activation; acute/relievable ER stress)	GLUT1, HK2 ↑ (glycolysis ↑); autophagy/mitophagy → mitochondrial quality control	▲ Consistent with effector function / early expansion	(8, 100, 101)
	Macrophages (IL-4 polarization; chronic/unrelieved)	ATF4 → PSAT1 ↑ → serine ↑, α-ketoglutarate ↑ → JMJD3-dependent epigenetic remodeling	▼ Stabilizes immunosuppressive M2 phenotype	(106)
	TAM / microglia (GBM; chronic/unrelieved)	ATF4–GLUT1 axis consistent with increased glucose uptake / glycolysis	▼ Consistent with myeloid suppressive metabolic state	(107)
	TAM (tumor tissue; chronic/unrelieved)	ARG1 ↑ → arginine depletion (polyamine/urea cycle); T-cell CD3ζ ↓, proliferation limited; PERK inhibition → M1-like	▼ Suppresses T cells; reversible with intervention	(108)
IRE1α–XBP1 (XBP1s/RIDD)	CD8^+^ TIL (chronic/unrelieved; hypoxia/glucose deprivation)	GLUT1 ↓, SLC38A2 ↓ → glucose/glutamine uptake ↓; IFN-γ ↓, respiratory chain activity ↓	▼ Consistent with exhaustion features	(14, 102, 103)
	TAM/M2 (chronic/unrelieved)	Tends toward FAO/OXPHOS; inhibiting IRE1–XBP1 → M2→M1 repolarization, TNF-α/IL-12 ↑	▼ Suppressive program	(109)
6;/’	Myeloid (GBM; hypoxia)	XBP1 cooperates with HIF-1α, stabilizing M2-like myeloid cells	▼ Suppresses antitumor responses	(14)
	MDSC (driven by tumor-secreted protein/lipid)	IRE1α–XBP1 activation → IgM and cytokines ↑ → MDSC infiltration ↑	▼ Promotes immunosuppression	(111, 110),
	MDSC (tumor cholesterol axis; hypoxia)	XBP1 binds HMGCR/HMGCS1 in tumor cells → cholesterol secretion ↑; exogenous cholesterol → STAT3-P / ROS ↑ (MDSC activation)	▼ Reinforces immunosuppression	(7)
	Tumor-associated DC (ROS/lipid peroxidation by-products; chronic/unrelieved)	Lipid metabolites → ER stress → XBP1s-driven lipogenic reprogramming → lipid droplets ↑ → cross-presentation failure; DC-Xbp1^-^/^-^ reverses	▼ Antigen presentation impaired	(4)
	Th17 (hypoxia/glucose deprivation induces XBP1 splicing)	XBP1s promotes Th17 differentiation; TUDCA reversible; EAE disease course mitigated	↔ Context-dependent (net effect in tumors to be determined)	(104, 105),
Other UPR regulators	Gp96 (CD4^+^ T; early after TCR engagement)	Ca²^+^ mobilization and glycolytic flux support early clonal expansion (when present/controlled)	▲ Supports activation (deficiency is detrimental)	(100)
	BAT3 (tumor-derived DC; selectively downregulated)	IRE1α program ↑, metabolic reconfiguration, glucocorticoid synthesis ↑ → impaired priming	▼ Inhibits T-cell priming	(112)

## 6 Immune cell metabolites reprogram tumor phenotypes

### 6.1 Metabolic reprogramming of immune cells shapes tumor immune evasion

Tumor immune evasion is a highly complex biological process involving the coordinated interaction of various cellular components and signaling pathways within the TIME. Metabolic reprogramming of immune cells contributes to the remodeling of their phenotype and effector functions in the tumor microenvironment.

#### 6.1.1 PD-1–associated immunometabolic networks

Programmed death-1 (PD-1), a hallmark of T cell exhaustion, is tightly regulated by the metabolic landscape of the TIME (113). Recent findings have unveiled intricate connections between metabolic stress and PD-1–mediated immune escape. In CD4^+^ T cells, methionine deprivation leads to AMPK downregulation and enhanced PD-1 expression, contributing to functional impairment and promoting tumor immune evasion (114). In macrophages, the IRE1–XBP1 axis drives glycolytic reprogramming and suppresses fatty acid oxidation (FAO), favoring polarization toward an M1-like phenotype and enhancing the therapeutic efficacy of PD-1 blockade (109). In contrast, the PERK–ATF4–PSAT1 pathway in melanoma promotes serine synthesis and α-ketoglutarate (α-KG) accumulation, stabilizing M2 macrophage polarization. This immunosuppressive metabolic state limits T cell infiltration, facilitates tumor growth, and attenuates PD-1 immunotherapy responsiveness (106).

#### 6.1.2 PD-1–independent pathways of immunosuppression

Beyond PD-1–mediated checkpoint control, immune cell metabolism is profoundly reprogrammed within the TIME, fostering immune dysfunction through diverse mechanisms. In glioblastoma, monocyte-derived macrophages exhibit PERK–ATF4–dependent upregulation of GLUT1, promoting glycolytic flux and lactic acid accumulation. Lactate acts as an epigenetic modulator by inducing histone lactylation at the IL-10 promoter, thereby enhancing IL-10 secretion and suppressing antitumor T cell responses (115). In parallel, XBP1s activation in CD4^+^ T cells drives ER-associated degradation (ERAD) of glutamine transporters, limiting glutamine uptake and mitochondrial respiration, and ultimately impairing IFN-γ production and effector function (103).

Lipid metabolic stress further exacerbates immune suppression across multiple cell types. DC dysfunction in ovarian cancer—driven by XBP1-mediated lipid accumulation—reduces T cell priming, directly contributing to immune evasion (4). Macrophages internalize oxidized low-density lipoprotein (oxLDL) through CD36, activating IRE1α and ATF6 arms of the UPR and promoting the formation of lipid-laden, immunosuppressive macrophages (116). Meanwhile, tumor-induced endoplasmic reticulum stress suppresses TAGLN2 in CD8^+^ T cells, impairing fatty acid uptake and mitochondrial fitness, thereby dampening cytotoxic activity (117). Macrophages internalize oxidized low-density lipoprotein (oxLDL) through CD36, activating IRE1α and ATF6 arms of the UPR and promoting the formation of lipid-laden, immunosuppressive macrophages (116).

A distinct immunosuppressive population, termed TAN-1, exhibits elevated glycolytic activity consistent with the “reverse Warburg effect,” wherein stromal cell-derived metabolites are exploited by tumor cells to fuel their growth. Concomitantly, the PERK–eIF2α–ATF4–CHOP axis upregulates the glutathione-degrading enzyme ChaC1, reducing intracellular antioxidant defenses in DCs and further impairing their immunostimulatory function, leading to progressive T cell exhaustion. Collectively, these multi-layered metabolic rewirings—spanning glucose, amino acid, and lipid metabolism—reshape the immune microenvironment in favor of tumor progression, independent of canonical PD-1 signaling pathways (118).

Together, these findings delineate a complex immunometabolic network involving glucose, amino acid, and lipid pathways that converge to orchestrate tumor immune escape.

### 6.2 Metabolic reprogramming of immune cells promotes tumor stemness

Tumor stemness—defined by the self-renewal capacity and high tumorigenicity of cancer stem cells (CSCs)—drives tumor progression, metastasis, recurrence, and therapeutic resistance. While intrinsic pathways such as WNT, Notch, and Hedgehog have been well-characterized in sustaining CSCs, recent findings reveal that immune cell–derived metabolic cues also critically influence stemness programming.

CD8^+^ T cell–derived interferon-γ (IFNγ) has been shown to suppress FGF2 expression and inhibit pyruvate kinase M2 (PKM2), a key glycolytic enzyme in tumor cells. This results in reduced NAD^+^ availability and promotes β-catenin stabilization, thereby enhancing the transcription of stemness-associated genes and augmenting tumor-initiating potential (119). In parallel, mannose metabolism within metabolically reprogrammed T cells fuels the hexosamine biosynthesis pathway, generating UDP-GlcNAc to activate O-GlcNAc transferase (OGT). OGT-mediated glycosylation of β-catenin stabilizes the protein and activates WNT signaling, further inducing expression of stemness regulators such as SOX2 and OCT4 (120).

Lactate, a glycolysis-derived metabolite enriched in the TIME, also contributes to the maintenance of CSC identity. CSCs exhibit enhanced lactate uptake, which supports mitochondrial metabolism, increases acetyl-CoA production, and promotes histone acetylation at oncogenic loci including MYC, sustaining stem-like phenotypes (121). Intriguingly, lactate may also bolster CD8^+^ T cell–mediated immunity: in murine MC38 tumor models, subcutaneous injection of sodium lactate, but not glucose, suppressed tumor growth via a CD8^+^ T cell–dependent mechanism—suggesting a context-dependent role of lactate in shaping both stemness and immune activation (122).

Moreover, tumor-associated macrophages (TAMs) serve as regulators of redox dynamics within CSC niches. Nanoparticle uptake by TAMs alters lysosomal iron handling and elevates local iron levels, rendering CD44^+^ CSCs susceptible to ferroptosis. Inhalable nanotherapy targeting TAM–CSC crosstalk has shown promise in reducing CSC populations and restraining early-stage lung tumor progression in preclinical models (123).

Collectively, these studies highlight the synergistic roles of metabolic reprogramming and ER stress signaling in shaping tumor stemness and suggest novel therapeutic avenues targeting CSCs through the metabolic-immune interface.

### 6.3 Molecular mechanisms by which immune cell metabolites promote tumor EMT, invasion, and metastasis

Immune cell metabolism within the TIME plays a pivotal role in promoting EMT and metastasis. In lung cancer, lactate derived from glycolytic immune cells directly induces Snail expression, facilitating EMT and tumor invasion. Tumor-associated macrophages (TAMs), through enhanced glycolysis, accumulate lactate and succinate, which activate O-GlcNAc transferase (OGT) and promote O-GlcNAcylation of cathepsin B, enhancing its secretion and matrix remodeling capacity (124, 125).

In parallel, metastasis-associated macrophages (MAMs) upregulate CD36, a scavenger receptor that facilitates uptake of long-chain fatty acids from tumor-derived vesicles. This lipid loading supports M2 polarization and metastatic colonization, particularly in the liver. Elevated glycolytic activity in TAMs also enhances cathepsin B–mediated metastasis and chemoresistance (124).

**Figure 4:**
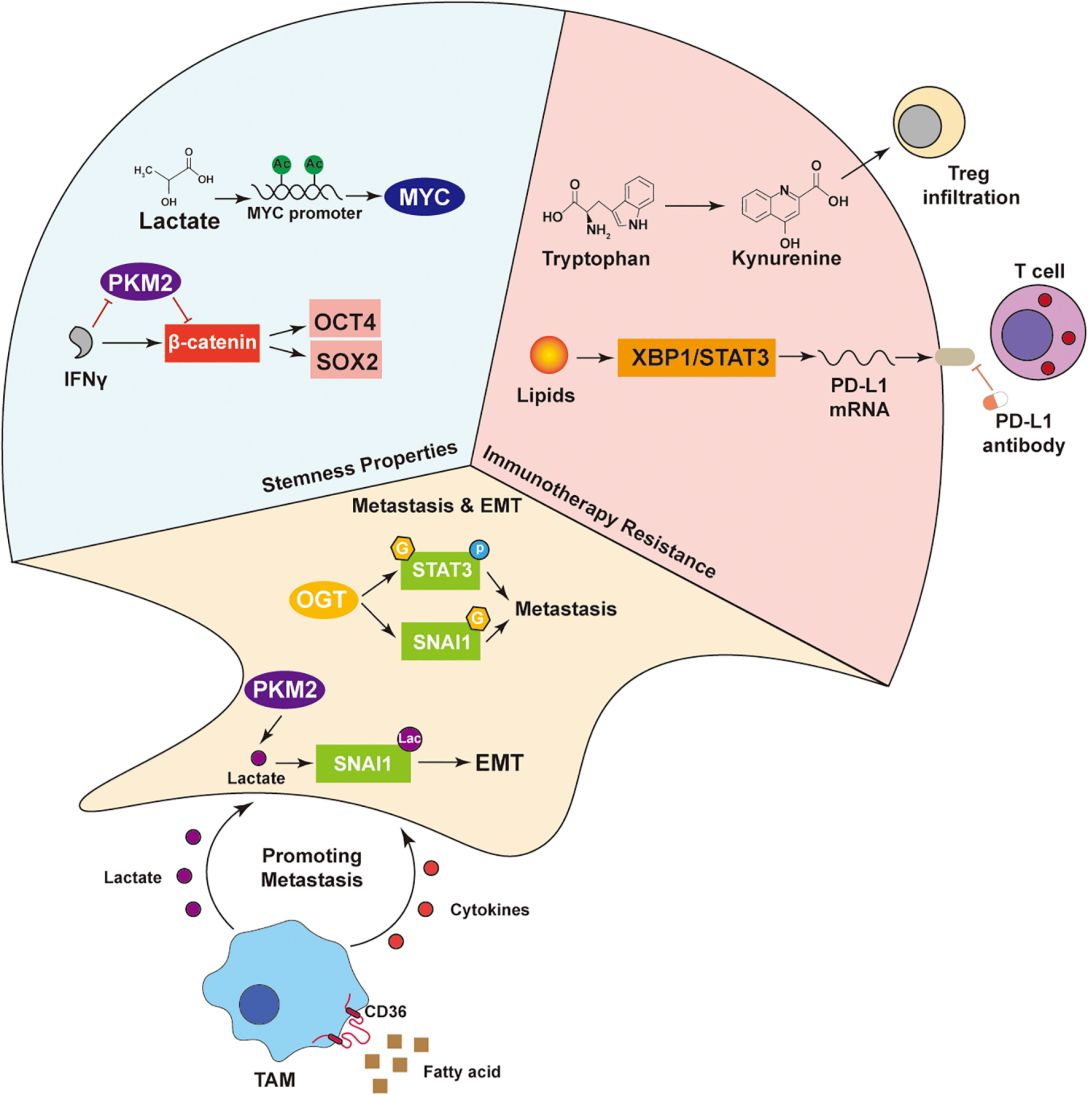
Multi-mechanism network driving tumor malignant phenotypes. Lactate maintains tumor stemness by activating OCT4 and SOX2 via MYC, with IFNγ enhancing this through the PKM2–β-catenin axis. In immunotherapy resistance, tryptophan-derived kynurenine promotes Treg infiltration, while lipids induce PD-L1 expression via XBP1/STAT3. SNAI1 drives EMT and metastasis. TAMs uptake fatty acids via CD36 and secrete cytokines and lactate, supporting tumor progression. These interactions highlight the crosstalk among metabolism, signaling, and the immune microenvironment in malignancy.

Together, these findings highlight how metabolically reprogrammed immune cells remodel the metastatic niche by coupling glycolytic and lipid metabolism with protease activation, thereby driving tumor dissemination (**Figure 4**).

## 7 Targeting key vulnerabilities via ER stress, metabolism, and immunometabolic modulation

### 7.1 Anti-tumor effects of PERK/eIF2α pathway inhibitors

Research demonstrates that in pancreatic ductal adenocarcinoma, the PERK/eIF2α phosphorylation inhibitor GSK2606414 significantly attenuates the Warburg effect, manifested by reduced glucose uptake and decreased lactate production, thereby inhibiting tumor growth and prolonging survival (25). Compared to wild-type KRAS cell lines (H1299, H1703), GSK2606414 exhibits more pronounced inhibitory effects on the colony-forming capacity of KRAS G12C mutant lung adenocarcinoma cells (H358, H23) (126). Furthermore, this drug demonstrates stronger cytotoxic effects on drug-resistant human breast cancer cell lines MCF-7-EpiR and MCF-7-TaxR than on parental MCF-7 cells (127). Notably, GSK2606414 enhances the susceptibility of human head and neck squamous cell carcinoma cells (HN5 and FaDu) to reovirus infection through an ATF4-dependent mechanism, while also increasing the sensitivity of human glioblastoma U87 cells to the combined treatment of simvastatin-temozolomide (128–130).

### 7.2 Clinical application prospects of IRE1α inhibitors

The small molecule IRE1α inhibitor MKC8866 is currently in clinical trials. In prostate cancer, this agent not only inhibits cancer cell growth but also enhances anti-PD-1 therapeutic effects by remodeling the TIME (131). In glioma models, MKC8866 as an adjuvant therapy significantly improves the efficacy of radiochemotherapy, evidenced by increased intratumoral necrotic areas and prolonged survival (132). For ovarian cancer and rhabdomyosarcoma, MKC8866 effectively reverses chemoresistance by inhibiting the IRE1α pathway, markedly reducing cell viability and proliferation capacity (133, 134).

### 7.3 Clinical research progress of IDO1 inhibitors

Epacadostat, as a highly potent and selective IDO1 inhibitor, is currently undergoing Phase III clinical trials. In Phase I studies involving patients with advanced solid tumors, administration of ≥100mg twice daily (BID) reduced plasma kynurenine levels to those observed in healthy subjects (135). Preliminary analysis of the Phase III clinical trial (NCT03374488) for advanced urothelial carcinoma (UC) showed that in patients with unresectable locally advanced or recurrent/progressive metastatic UC who failed first-line platinum-based chemotherapy, the objective response rate (ORR) of epacadostat combined with pembrolizumab was numerically higher than that of pembrolizumab monotherapy (26.2% vs. 11.9%) (136). The ECHO-202/KEYNOTE-037 study results indicated that pembrolizumab combined with epacadostat was well-tolerated and demonstrated durable objective response evidence in patients with metastatic renal cell carcinoma (mRCC) (137). The KEYNOTE-679/ECHO-302 study results revealed that in previously untreated patients with locally advanced or metastatic renal cell carcinoma, pembrolizumab combined with epadostat showed similar response rates compared to sunitinib or pazopanib (138).

### 7.4 Metabolic intervention therapeutic strategies

The glutaminase inhibitor CB-839 has become a research focus due to its mechanism targeting tumor cell metabolic characteristics (139). In lung cancer, CB-839 delays cellular metabolism and energy production by inhibiting the TCA cycle and glutamine-dependent biosynthetic pathways, inducing cells into a dormant state (140). For KRAS-mutant ovarian cancer cells, the combination of metformin and CB-839 significantly reduces aerobic oxidation capacity and proliferative activity (141). In preclinical models of PIK3CA-mutant colorectal cancer (CRC), the combined treatment of CB-839 and 5-FU significantly inhibited tumor growth through NET inhibition mechanisms (142).

### 7.5 Lactate metabolism regulation and immune microenvironment

The LDHA inhibitor GNE improves immunosuppressive status by inhibiting lactate dehydrogenase A (LDHA) activity and reducing lactate (LA) secretion in the TIME (143). In melanoma models, treatment with GNE-140-containing hydrogel significantly increased intratumoral infiltration of CD3^+^CD8^+^ T cells, inhibited tumor growth, and enhanced anti-tumor immune responses (144).

### 7.6 Immune checkpoint regulation and metabolic intervention

PVR/CD155 is overexpressed in various malignancies and mediates immune escape by binding to T cell immune receptors. Studies found that acetate downregulates PVR/CD155 expression levels by inhibiting the PI3K/AKT signaling pathway, thereby enhancing the anti-tumor activity of CD8^+^ T cells (145).

### 7.7 Metabolic regulation of CAR-T cell therapy

**Figure 5:**
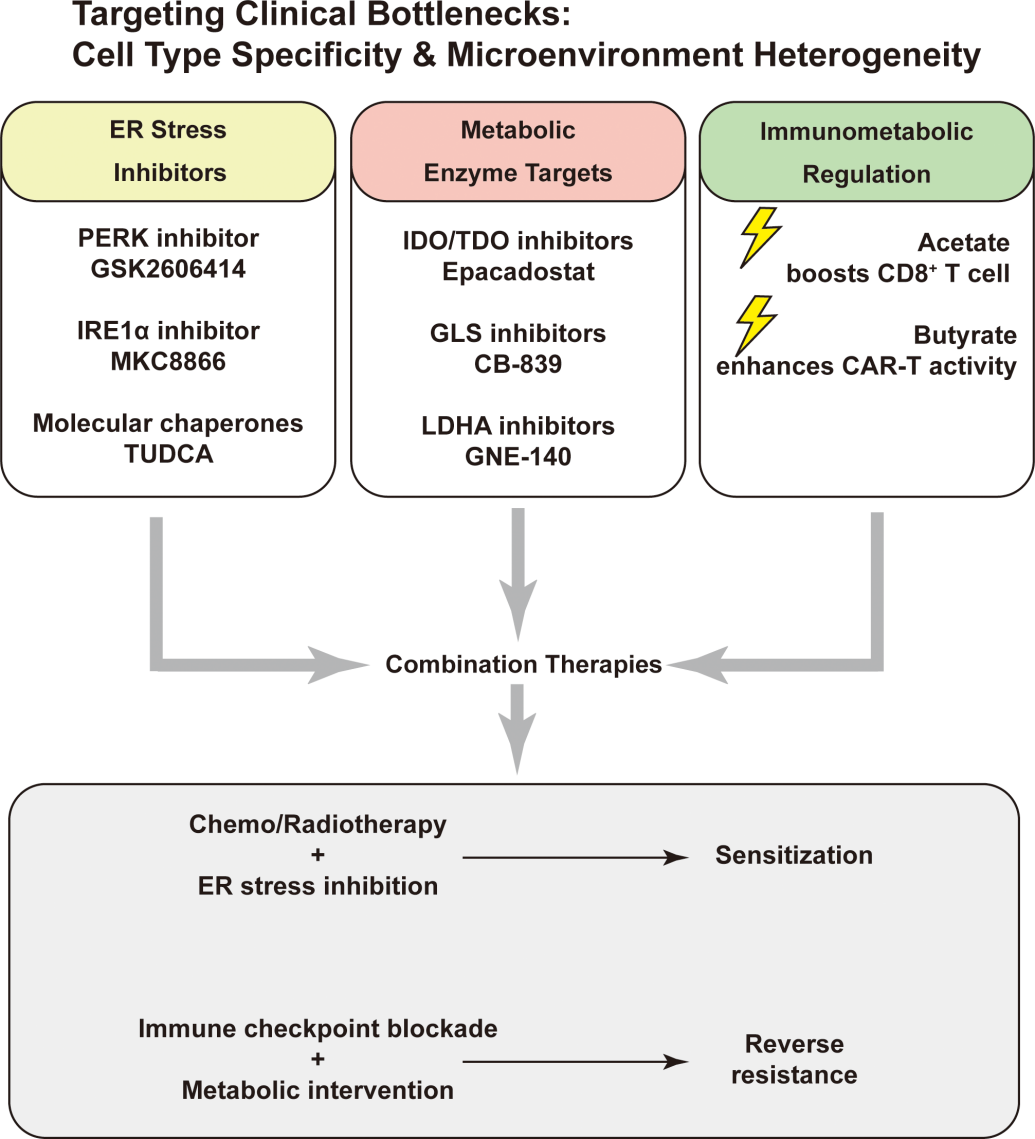
Targeting key vulnerabilities via ER stress, metabolism, and immunometabolic modulation. Potential therapeutic targets include ER stress pathways (PERK, IRE1α), metabolic enzymes (IDO/TDO, GLS, LDHA), and immunometabolic regulators (acetate, butyrate). Combination strategies may enhance chemo/radiosensitivity or overcome immune resistance.

Genetic modification to express chimeric antigen receptors (CAR) enables T cells to specifically recognize tumor antigens. Research by Luu et al. (70) demonstrated that short-chain fatty acids (such as valerate and butyrate) can enhance the expression of cytotoxic effector molecules in ROR1-specific CAR T cells, improving their anti-tumor efficacy. Notably, valerate similarly enhances human CAR T cells, indicating the potential application value of microbial metabolites in tumor immunotherapy. In colorectal cancer models, butyrate significantly improved the efficacy of anti-PD-1 therapy by upregulating TLR5 expression on the surface of CD8^+^ T cells (146) (**Figure 5**).

## 8 Summary and discussion

The ER stress response is no longer viewed as a mere adaptive reaction to proteostatic imbalance, but as a pivotal orchestrator of cellular fate in the tumor microenvironment. This integrated ‘ER stress–metabolism–immunity’axis, by coupling nutrient sensing to immune remodeling, presents actionable targets for overcoming immunotherapy resistance and enhancing antitumor responses. The convergence of ER stress with glucose, lipid, and amino acid metabolism drives not only intrinsic tumor cell plasticity but also extrinsic immunosuppressive remodeling via key metabolites such as lactate and lipid mediators. Likewise, the reciprocal metabolic rewiring of immune cells under ER stress further reinforces a vicious cycle that promotes tumor heterogeneity and immune tolerance.

Here, we establish metabolism as the operational hub linking endoplasmic reticulum stress to antitumor immunity. We present a branch-resolved, metabolite-centric framework that maps the PERK/eIF2α/ATF4 arm, the IRE1α/XBP1 arm including RIDD, and ATF6 to discrete circuits in glucose metabolism, in lipid and sterol metabolism including sphingolipids, and in amino-acid metabolism, and we connect those circuits to defined immune endpoints such as antigen presentation and cross-priming, CD8^+^ T-cell exhaustion or fitness, NK cytotoxicity, and TAM or MDSC polarization. Prior reviews established the importance of ER stress but were largely organized by stress inducers and signaling or by immune-intrinsic effects. Compared with Nature Reviews Cancer (3) (2020), which catalogues tumor-microenvironment stressors and oncogenic crosstalk with broad immune consequences, we formalize the alignment between each UPR arm, its matched metabolite circuits, and immune endpoints, and we provide a decision framework for combination strategies and biomarker use rather than re-listing inducers. Compared with Trends in Cancer (2022) (5), which is cell-type centered on ER-stress-driven immunosuppression and vulnerabilities, we reorganize by metabolic modules, incorporate bidirectional crosstalk between tumor and immune metabolism, and derive combination hypotheses with graded levels of evidence. Compared with Nature Reviews Immunology (99) (2023), which emphasizes immune-intrinsic UPR and pattern-recognition or cytokine pathways, we place metabolism first and anchor dendritic-cell lipid handling and antigen-presentation failure, T-cell metabolic fitness and exhaustion, and myeloid polarization in specific metabolic nodes with clear stratification and pharmacodynamic readouts. We also integrate 2024–2025 advances in **Box 1** that give this framework clinical traction, including lactate/histone-lactylation–driven myeloid suppression in glioblastoma, tumor-released CNS-enriched metabolites that disrupt the T-cell immunological synapse, PD-L1–coupled metabolic rewiring that potentiates glutamine-pathway inhibition, SLC3A2-mediated lysine sequestration that limits T-cell fitness, and redox-axis diversion (glutamate→glutathione) that restores MHC-I antigen presentation; we further highlight nutrient/cofactor competition in the TIME (e.g., DHDH-linked D-xylose products and vitamin B6 limitation) that constrains NK and T-cell efficacy. Key points and clinical implications are summarized in **Box 2**, supporting co-targeting of UPR, metabolism, and immunity within defined metabolic modules.

Box 1 **2024–2025 updates and how they shift clinical thinking.**

Distinct from the above reviews, we integrate late-breaking studies (2024–2025) that pin immunometabolic control to concrete ER-stress/UPR programs and suggest actionable choices: (i) lactate and histone lactylation emerge as programmatic drivers of myeloid suppression under ER stress in glioblastoma, where PERK–HIF-1α–coupled glycolysis raises lactate; a pragmatic path is LDH or lactate or lactylation-directed strategies with UPR-branch modulators, with tumor lactate and lactylation load guiding selection (107, 122); (ii) a defined CNS-enriched metabolite released by tumor cells impairs the T-cell immunological synapse, arguing for metabolite interception or transporter blockade combined with UPR control in synapse-defective tumors (91); (iii) PD-L1–targeted metabolic rewiring heightens the antitumor effect of glutamine-pathway inhibition, consistent with PERK/ATF4-regulated amino-acid stress, supporting trials of glutamine inhibitors plus immune modulation with ATF4-linked signatures as companion markers (92); (iv) SLC3A2-mediated lysine uptake by cancer cells limits T-cell fitness in hepatocellular carcinoma, nominating neutral amino-acid transporter targeting or nutrient restoration together with checkpoint blockade in lysine-scarce contexts (90); (v) blocking glutamate-to-glutathione flux increases MHC-I antigen presentation in colorectal cancer cells, supporting redox-axis interference with checkpoint blockade where antigen display is poor, with GSH/GSSG balance and processing signatures informing use (96); and (vi) newly identified metabolites linked to ER stress and the UPR compromise antigen processing and immune-synapse integrity, including DHDH-driven D-xylose products that weaken T-cell synapses, a CNS-enriched tumor metabolite that blocks T-cell engagement, and vitamin B6 competition that limits NK cytotoxic synapses; a pragmatic path is metabolite interception or transport blockade or cofactor restoration combined with UPR modulation, with synapse integrity, DHDH activity, and vitamin B6 status guiding selection (57, 73, 91). Together these additions provide specific biomarker cues and combination templates not available in the 2021–2023 reviews, and they operationalize UPR–metabolism–immunity co-targeting within defined circuits.

Box 2 **Nuances and contradictions: context-dependent metabolite effects.**

(1) Lactate. (i) Endogenous high lactate (hypoxia/high glycolysis; acting on CD8^+^ T cells): Suppresses cytotoxic function by inhibiting pyruvate carboxylase and activating pyruvate dehydrogenase, reducing extracellular succinate, and blunting SUCNR1-dependent signaling and glycolytic activity (55); (ii) Myeloid cells (TAMs/monocyte-derived macrophages): Histone lactylation upregulates IL-10, indirectly suppressing T-cell responses (see main-text citations) (116); (iii) Exogenous/low-dose lactate (e.g., MC38; acting on CD8^+^ T cells): Enhancing — under exogenous, low–moderate dose, short exposure, with preferential T-cell lactate uptake, adequate oxygen/respiration, and early activation/expansion, lactate can serve as a carbon source (→ pyruvate → mitochondria), improving metabolic fitness/stemness and antitumor activity (123); Inhibitory — at high dose or prolonged exposure, with high local lactate/low pH, preferential action on myeloid cells (lactylation→IL-10), or further depression of T-cell glycolysis (e.g., PC inhibition), the net effect is suppressive (55).

(2) SCFAs. (i) Acetate (glucose-restricted/stress contexts; ACSS2/ACSS1; TIL-CD8^+^): Via ACSS2, acetate elevates histone acetylation to enhance effector function; blocking tumor uptake increases acetate available to immune cells, and ACSS1 supports mitochondrial respiration and effector activity (68, 69).; (ii) Butyrate and propionate (colorectal cancer context): Can enhance CD8^+^ T-cell responses by inducing antigenicity remodeling linked to DNA damage (71). However, butyrate/propionate can also suppress antigen-specific CD8^+^ T-cell activation by reducing IL-12/IL-23 production by DCs (147); (iii) Microbiota-derived butyrate in ACT/CAR-T settings: Enhances antitumor efficacy, including via mTOR activation, increased cytokine production, and boosted CD8^+^ T-cell responses (70).

Targeting the “ER stress–metabolism–immunity” axis offers a promising avenue to recalibrate tumor immunometabolism and sensitize malignancies to existing or emerging therapies. Future research should aim to elucidate context-dependent ER stress signaling in distinct immune and tumor compartments, explore cell-type-specific vulnerabilities, and develop combinatorial strategies that leverage metabolic intervention to restore immune competence. Bridging these interdisciplinary domains will be critical for the next generation of precision immunometabolic cancer therapy.

Although this study systematically elucidates the regulatory mechanisms of the ER stress-metabolism-immunity axis, several methodological limitations warrant attention. Firstly, regarding experimental models, the current research primarily relies on immortalized cell lines and murine models, which exhibit significant shortcomings in recapitulating the native heterogeneity, metabolic characteristics, and microenvironmental interactions of human primary cells. Secondly, at the mechanistic level, the cell type-specific response patterns to ER stress remain uncharacterized, and these differences are crucial for precise therapeutic target selection. Lastly, current technical approaches lack the capacity for simultaneous monitoring of ER stress signaling and dynamic changes in local metabolic microenvironments. To address these critical scientific questions, future research could advance along three dimensions: First, the integration of high-throughput technologies such as spatial transcriptomics and single-cell metabolomics to systematically construct a spatiotemporal atlas of ER stress responses at single-cell resolution in human tumor microenvironments, with particular emphasis on elucidating their dynamic spatial correlations with key metabolic parameters. Second, establishing patient-derived organoid-autologous immune cell 3D coculture systems that preserve the molecular signatures of primary cells and microenvironmental interaction networks, thereby providing more translationally relevant experimental platforms for preclinical research. Third, developing deep learning-based multi-omics integration algorithms to build cell type-specific predictive models of ER stress responses, incorporating multidimensional data including pathway activity, metabolic status, and drug sensitivity to optimize precision therapeutic strategies. These innovative research approaches will significantly enhance the clinical translation efficiency of fundamental discoveries and establish a solid theoretical foundation for developing ER stress-based personalized cancer therapies.
